# Hypoxemic Human Metapneumovirus Pneumonia in a Young Immunocompetent Man in the First Autumn After the Reclassification of Coronavirus Disease

**DOI:** 10.7759/cureus.76455

**Published:** 2024-12-27

**Authors:** Yoshihiro Kitahara, Yusuke Takayama, Goki Ushio, Shoshi Akieda, Toshiro Takafuta

**Affiliations:** 1 Department of Internal Medicine, Hiroshima City Funairi Citizens Hospital, Hiroshima, JPN

**Keywords:** acute respiratory failure, autumn, co-infection, haemophilus influenzae, human metapneumovirus, multiplex-nested polymerase chain reaction, pneumonia, reclassification of coronavirus disease, young immunocompetent adults

## Abstract

Although human metapneumovirus* *(hMPV) infection can induce severe symptoms in older adults or immunocompromised patients, it usually causes mild symptoms in young immunocompetent adults. The prevalence of hMPV infectious disease is highest during the late winter and early summer. We report a hypoxemic case of hMPV infection in a young immunocompetent man that occurred in the first autumn after the reclassification of coronavirus disease (COVID-19) from Class 2 to Class 5. A multiplex-nested polymerase chain reaction (MN-PCR) test using a sputum sample was useful for rapid detection of co-infection with hMPV and *Haemophilus influenzae (H. influenzae) *as causes of pneumonia and acute respiratory failure. In early October 2023, a 24-year-old healthy man without comorbidities visited our hospital with a fever, sore throat, and nasal congestion. The antigen test results for influenza and COVID-19 were negative. After 6 days, he presented with a productive cough and exertional dyspnea. He was immediately admitted to our hospital due to hypoxemia with a percutaneous arterial oxygen saturation of 90% breathing room air. Although bacterial pneumonia was suspected based on bilateral ground-glass opacities and infiltrative shadows on chest computed tomography (CT), the MN-PCR test using a nasopharyngeal swab indicated hMPV infection. On the 2^nd^ day of admission, the MN-PCR test using a sputum sample (Group 5 in the Geckler classification) indicated that hMPV and* H. influenzae *were the causative pathogens for pneumonia. We initiated oxygen supplementation and administered intravenous ceftriaxone sodium hydrate and oral azithromycin hydrate. The patient was discharged on the 7^th^ day of admission with improved pneumonic shadows and hypoxemia. hMPV infection can occur in any season in the post-COVID-19 era. Even in young immunocompetent adults, hMPV infection can cause pneumonia which may progress to acute respiratory failure with other bacterial co-infection. Conversely, it is clinically important to pay attention to the involvement of hMPV and other respiratory viruses in pneumonia cases that initially appear to be bacterial based on CT findings. MN-PCR test using a good-quality sputum sample is valuable not only for rapid detection of causative bacteria and viruses for pneumonia but also for identifying these co-infections.

## Introduction

Human metapneumovirus (hMPV), isolated in the Netherlands in 2001, is a negative-sense, single-stranded ribonucleic acid virus [1,2]. hMPV is classified into the genus *Metapneumovirus *of the sub-family *Pneumovirinae* in the family *Paramyxoviridae* [1,2]. hMPV is transmitted across all ages via droplet or contact infection [3,4]. hMPV breaks through the mucosal layer and primarily replicates in the airway epithelial cells located in the nasopharynx, trachea, bronchi, and lungs [5]. Further, hMPV directly injures these upper and lower airway epithelial cells and causes inflammatory changes leading to their remodeling [5]. Virions and the infected epithelial cells are eliminated by the humoral and cell-mediated immune systems, accompanied by a variable level of inflammatory response. These established immune systems are essential for hMPV clearance and anti-hMPV defense, and if the immune system is compromised, hMPV can persist in the airway epithelial cells or the airway lumen, leading to ongoing airway inflammation [5].

While hMPV infection can be asymptomatic, acute respiratory symptoms such as cough, nasal congestion, sore throat, wheezing, fever, and dyspnea mainly occur in children and older adults after an incubation period of 4-6 days [3,4]. In immunocompetent adult patients, these symptoms usually improve within 1 week [4]. However, hMPV infection can cause severe disease, presenting as pneumonia and acute respiratory failure in older adult patients, patients with comorbidities such as chronic obstructive pulmonary disease, and those who are immunocompromised such as patients with hematological malignancies [3].

The epidemic season of hMPV infectious disease is reported as March to June [2] or late winter to spring [6]. Although hMPV infectious disease can occur sporadically in all seasons, the incidence rate is considered low during autumn in the Northern Hemisphere, regardless of the hMPV genotype [6]. Recently, a remarkable decrease in the number of hMPV infections owing to the coronavirus disease (COVID-19) pandemic has been reported [7]. A possible reason for this phenomenon is that hMPV is an enveloped virus, and it is likely to be influenced by nonpharmaceutical interventions, such as social distancing, mask-wearing, and hand hygiene. Moreover, a change in the seasonal trend of hMPV infections after the COVID-19 pandemic has also been reported [7]. In Korean children, the detection rate of hMPV decreased to 0% from the 2^nd^ half of 2020 to the 1^st^ half of 2022. It increased again with the full relaxation of nonpharmaceutical interventions in April 2022 and showed an unusual increase in autumn 2022 [7].

In Japan, the classification of COVID-19 was changed from Class 2 to Class 5 under the Infectious Diseases Control Law on May 8, 2023 [8], and the Japanese government stopped requiring uniform basic infection control measures for the public. Thereafter, cases of hMPV began rising in July 2023, according to a report by the National Institute of Infectious Diseases [9].

Therefore, careful monitoring of the increase in hMPV infection is important in the post-COVID-19 era. However, in Japan, qualitative antigen tests for hMPV infection are covered by medical insurance only for children <6 years old [10], making diagnosis in patients ≥6 years old difficult in clinical settings.

Recently, a multiplex-nested polymerase chain reaction (MN-PCR) test using the microarray method has been widely performed for the diagnosis of acute respiratory infectious diseases [11]. MN-PCR test which uses a nasopharyngeal swab and a dedicated test kit (The BioFire^Ⓡ^ Respiratory 2.1 (RP2.1) Panel (BioMérieux Japan Ltd.), hereinafter called “respiratory panel test”), can simultaneously detect the nucleic acids of multiple target pathogens (18 viruses, 2 bacteria and 2 atypical bacteria) [12]. The respiratory panel test can be used regardless of the patient’s age, and test results are available within approximately 1 hour [12].

We herein report a case of hMPV infectious disease that occurred in the first autumn after the reclassification of coronavirus disease and progressed to bilateral pneumonia and acute respiratory failure despite the patient being a young immunocompetent man. Without the MN-PCR test, we could not have suspected hMPV infection and would have diagnosed *Haemophilus influenzae* (*H. influenzae*) pneumonia alone based on computed tomography (CT) findings and sputum culture test results.

## Case presentation

A 24-year-old healthy man without comorbidity developed a fever, sore throat, and nasal congestion in early October 2023. He was a current smoker with a Brinkman index of 60 and his alcohol consumption was 100 mL of whiskey per day. Nothing in his medical history suggested a primary immunodeficiency. He was unvaccinated for severe acute respiratory syndrome coronavirus 2 (SARS-CoV-2) and had no history of COVID-19. He had no significant medical history except for an allergy to pistachios, which caused a skin rash.

The patient visited our hospital the next day (2^nd^ day of onset). No crackles or wheezing were observed on auscultation. The antigen test results for influenza and COVID-19 were negative. Accordingly, the patient was diagnosed with a common cold and treated with L-carbocisteine, ambroxol hydrochloride, and tipepidine hibenzate.

Subsequently, the patient developed a productive cough and exertional dyspnea and visited our hospital 6 days later (8^th^ day of onset). He was immediately admitted due to hypoxemia with a percutaneous arterial oxygen saturation (SpO_2_) of 90% breathing room air. The patient had a clear level of consciousness. His height and weight were 171.1 cm and 59.0 kg, respectively, with a lean body and a body mass index of 20.2 kg/m². He had a body temperature of 37.1°C, a blood pressure of 109/69 mmHg, a pulse rate of 60 beats/min with a regular rhythm, and a respiratory rate of 24 breaths/min. On auscultation, his respiratory sounds revealed bilateral coarse crackles and his heart sounds showed fixed splitting of sound I in the second intercostal space on both sides of the sternum. No peripheral skin lesions or edema were present.

**Figure 1:**
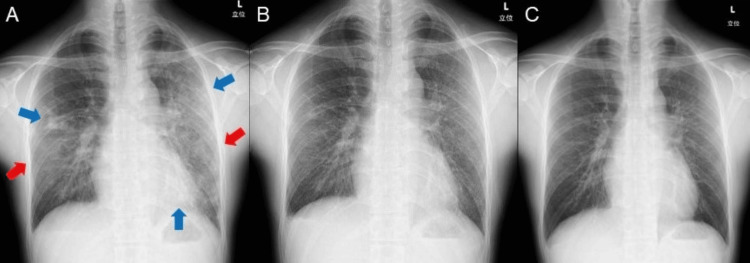
Time-course change in chest radiograph findings. (A) Chest radiograph obtained on the day of admission showing extensive ground-glass opacities (red arrows) and infiltrative shadows (blue arrows) in both lung fields; (B) Chest radiograph obtained on the 3^rd^ day of admission showing improvement in the pneumonic shadows; (C) Chest radiograph obtained on the 7^th^ day of admission showing further improvement in the pneumonic shadows.

A chest radiograph obtained on the day of admission showed extensive ground-glass opacities and infiltrative shadows in both lung fields (Figure 1A).

**Figure 2:**
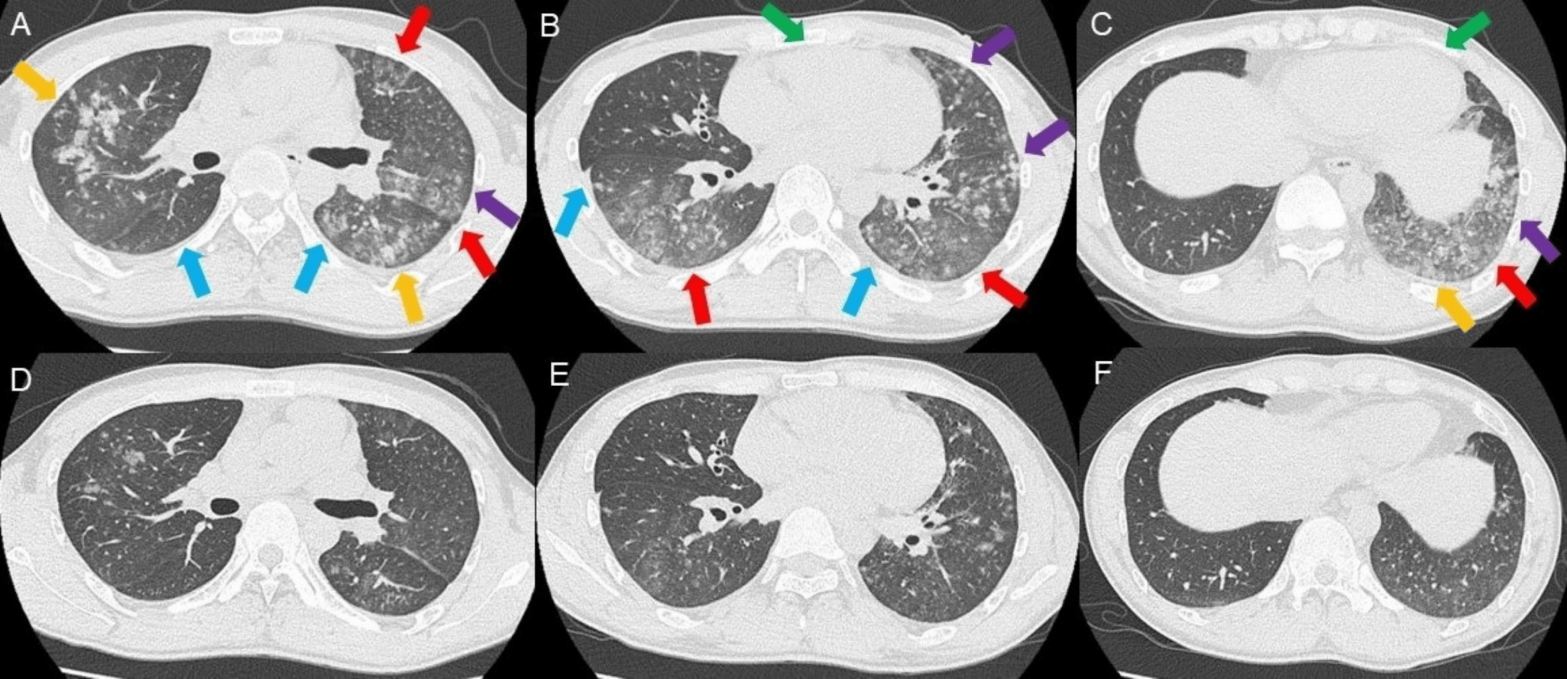
Time-course change in findings of computed tomography (CT). (A–C) CT on the day of admission. Ground-glass opacities (red arrows), granular shadows (purple arrows), and nodular infiltrative shadows (orange arrows) in both lung fields. Although there is a small amount of bilateral pleural effusions (light blue arrows), cardiac enlargement is not observed (green arrows); (D–F) CT on the 3^rd^ day of admission showing improvement in the pneumonic shadows in both lung fields and a decrease in bilateral pleural effusions.

CT revealed ground-glass opacities, granular shadows, and nodular infiltrative shadows in both lung fields, suggestive of bacterial pneumonia (Figures 2A-2C). Although we observed a small amount of bilateral pleural effusions, no cardiac enlargement was noted.

Laboratory tests revealed an increased white blood cell count (WBC) of 9,100 cells/μL (normal range: 3,300-8,600 cells/μL), with normal fractions: neutrophils 68.5%, eosinophils: 2.4%, basophils: 0.5%, monocytes: 6.3%, and lymphocytes: 22.3%. C-reactive protein (CRP) levels were elevated at 4.47 mg/dL (normal range: <0.14 mg/dL), and lactate dehydrogenase (LDH) levels were elevated at 287 U/L (normal range: 124-222 U/L).

The particle agglutination method revealed that the patient was negative for immunoglobulin M antibodies against *Mycoplasma pneumoniae* (<40-fold) and *Chlamydophila pneumoniae* (0.0). The erythrocyte sedimentation rate slightly increased to 16 mm/hour. Based on the normal levels of Krebs von den Lungen-6 (229 U/mL), β-D glucan (7 pg/mL), N-terminal pro-brain natriuretic peptide (18 pg/mL), proteinase 3-anti-neutrophil cytoplasmic antibody (ANCA) (0.6 IU/mL), and myeloperoxidase-ANCA (0.3 IU/mL), and negative results for anti-*Trichosporon asahii* antibody (0.00 corrected absorbance index), the possibility of acute interstitial pneumonia, *Pneumocystis jirovecii* pneumonia, acute heart failure, ANCA-associated vasculitis, and summer‐type hypersensitivity pneumonitis was ruled out. Urinary antigen tests for *Streptococcus pneumoniae* and *Legionella pneumophila* serogroup 1 were negative. To completely rule out influenza and COVID-19, we performed a respiratory panel test using a nasopharyngeal swab [12]. While the respiratory panel test showed negative results for influenza virus A, influenza virus B, and SARS-CoV-2, it showed a positive result for hMPV and we diagnosed with hMPV infection.

His sputum was slightly yellowish and judged to be of good quality according to Group 4 in the Geckler classification, an index for assessing microscopic sputum quality. In addition to the sputum culture test, we concurrently performed a respiratory panel test on a trial basis for our reference, using the remainder of the sputum sample. The respiratory panel test showed a positive result for hMPV, suggesting a diagnosis of hMPV pneumonia. Intravenous ceftriaxone sodium hydrate (CTRX) (2 g/day) and oral azithromycin hydrate (AZM) (500 mg/day for 3 days) were administered based on the possibility of bacterial co-infection. The patient’s acute respiratory failure was treated with oxygen supplementation at 2 L/min via a nasal cannula, and his SpO_2_ was maintained at 96-97%.

On the 2^nd^ day of admission (9^th ^day of onset), we performed the MN-PCR test which uses a better-quality sputum sample (Group 5 in the Geckler classification) and a dedicated test kit The BioFire^Ⓡ^ FilmArray^Ⓡ^ Pneumonia (PN) Panel (BioMérieux Japan Ltd.), hereinafter called “pneumonia panel test”), with permission from the hospital to rapidly identify the causative pathogens for pneumonia [13]. The pneumonia panel test can detect the nucleic acids of 15 bacteria with 7 antimicrobial resistance genes, 3 atypical bacteria, and 8 viruses [13], and this test was positive for hMPV and *H. influenzae* (10^5^ copies/mL). We obtained a positive result for *H. influenzae* in the sputum culture test on the 4^th^ day of admission (11^th^ day of onset). Based on these results, we concluded that the pneumonic shadows observed in the present case were due to a mixture of hMPV pneumonia and *H. influenzae* pneumonia.

After the initiation of antibiotics treatment, the fever did not recur. Productive cough and exertional dyspnea gradually improved with an increase in SpO_2_ of 97% breathing room air. Laboratory tests revealed a decrease in WBC (7,900 cells/μL), serum CRP (1.41 mg/dL), and LDH (204 U/L) levels. Although watery diarrhea occurred on the 2^nd^ day of admission (9^th^ day of onset), it was promptly alleviated by oral probiotics. On the 3^rd^ day of admission (10^th^ day of onset), a chest radiograph and CT revealed improvement in pneumonic shadows and a decrease in bilateral pleural effusions (Figure 1B and Figures 2D-2F).

The patient was discharged on the 7^th^ day of admission (14^th^ day of onset) after confirming further improvement in the pneumonic shadows on the chest radiograph (Figure 1C) and laboratory findings indicating a further decrease in the serum CRP (0.17 mg/dL) and LDH (164 U/L) levels. The patient refused permission to perform a human immunodeficiency virus antigen test. Two days after discharge (16^th^ day of onset), we obtained the results of antimicrobial susceptibility tests, which showed that *H. influenzae* was susceptible to CTRX and clarithromycin, a macrolide antibiotic like AZM.

## Discussion

Generally, it is presumed that the first hMPV infection occurs during childhood in most individuals [4,14]. Ebihara et al. reported that the hMPV immunoglobulin G (IgG) antibody prevalence rate was 76.7% among 2-5-year-olds, 92.9% among 5-10-year-olds, and 100% in individuals over 10 years old [14]. Conversely, there were substantial inter-individual differences in the hMPV IgG levels [14]. We consider that even within the same generation, hMPV IgG levels can differ according to the timing of infection, the ability to produce IgG, and the ability to maintain IgG levels.

The anamnestic immune response induced by hMPV infection is relatively weak, which may explain why people can develop repeated hMPV infectious diseases throughout their lives [5]. From a humoral immune system perspective, we speculate that hMPV infection can recur even in immunocompetent individuals if their hMPV IgG levels are low, or if a new hMPV genotype is encountered even in the presence of high hMPV IgG levels [4,15]. Conversely, we speculate that hMPV IgG levels gradually increase and maintain high levels due to repeated hMPV infection with various genotypes, resulting in milder disease severity in young immunocompetent individuals through the enhancement of the immune system against hMPV infection.

Therefore, the fact that hMPV infection caused pneumonia and acute respiratory failure in a healthy young immunocompetent man is an unusual clinical course and a unique aspect of the present case. We consider two possible reasons for this phenomenon. Firstly, he had not been previously exposed to this hMPV genotype, and insufficient immune protection resulted in severe disease. Secondly, regardless of the hMPV genotype, his total IgG levels against hMPV could have decreased due to the reduced opportunity for hMPV infection, given the extensive wearing of masks during the COVID-19 pandemic.

As far as we investigated through a personal questionnaire from BioMérieux Japan Ltd., as of April 2024, the respiratory panel test (original price: 686,400 Japanese yen per 30 test kits) is covered by Japanese medical insurance (13,500 Japanese yen per test) [12], while the pneumonia panel test (original price: 722,700 Japanese yen per 30 test kits) is not approved [13]. Therefore, it is difficult to use the pneumonia panel test in daily clinical settings, and most patients with positive results on the respiratory panel test using nasopharyngeal swabs and pneumonic shadows are clinically diagnosed with hMPV pneumonia. As respiratory and pneumonia panel tests use the same principle, further studies are warranted to expand the indications for respiratory panel tests using sputum and bronchoalveolar lavage fluid samples, similar to the pneumonia panel test [12,13].

As observed in the present case, ground-glass opacities and nodular infiltrative shadows are common CT findings in hMPV pneumonia [16]. However, these shadows are not necessarily specific to hMPV pneumonia. Moreover, because hMPV may occur as a co-infection with other viruses or bacteria [17,18], it is often difficult to judge whether these shadows on CT images represent hMPV pneumonia alone or the mixture of hMPV pneumonia and concurrent bacterial pneumonia or additional viral pneumonia. In the present case, *H. influenzae* infection was the other cause of pneumonia and might have exerted a greater influence on progression to hypoxemia than hMPV infection.

The pneumonia panel test using a sputum sample significantly assisted in determining that *H. influenzae* was the other causative pathogen for pneumonia on the 2^nd^ day of admission, suggesting that the empirical choice of CTRX and AZM for administration on the day of admission was probably appropriate. Conversely, *H. influenzae* was detected in the sputum culture test on the 4^th^ day of admission and obtained drug susceptibility test results 2 days after discharge (equivalent to the 9^th^ day of admission if he had still been admitted). Thus, although the pneumonia panel test is superior to the sputum culture test in the promptness for obtaining results, clinicians must note that not all viruses and bacteria detected by MN-PCR tests are necessarily causative pathogens, as the test results can be positive even if the pathogens are no longer present. Therefore, we should not omit simultaneous sputum culture tests to evaluate the credibility of causative bacteria obtained in the pneumonia panel test and to reconfirm the validity of the choice of antibiotics.

Viewed from a different angle, many cases of pneumonia may be diagnosed as bacterial pneumonia alone based on the CT findings and positive sputum culture, overlooking other viral co-infections. In the present case, although bacterial pneumonia can be treated with appropriate antibiotics, treatment of hMPV pneumonia typically requires waiting for natural improvement because no effective treatment exists. Consequently, the clinical course in the present case does not change regardless of the clinician’s recognition of hMPV co-infection. Nevertheless, detecting hMPV co-infection as a preceding respiratory infectious pathogen and/or as the other causative pathogen for pneumonia through respiratory and pneumonia panel tests is valuable for elucidating the disease state of pneumonia and contributing to the future developments in the epidemiology of acute respiratory infectious diseases.

Another characteristic feature of the present case is the occurrence of hMPV infection in autumn. As of October 2023, although sporadic cases of COVID-19 have been reported [19], there has been a remarkable monthly increase in the number of patients with influenza, surpassing those of patients with COVID-19 [20]. Moreover, as October is not typically a season with a high level of hMPV activity [2,6], we did not suspect hMPV infection based on medical interviews or CT findings in the present case. Although the hMPV detection rate increased markedly in Korean children in September 2022 [7], approximately 5 months after nonpharmaceutical interventions were fully relaxed, the hMPV detection rate had almost returned to the usual seasonal pattern in 2023. In Japan, careful attention should be paid to the annual changes in the number of hMPV infections after the reclassification of coronavirus disease, to assess whether hMPV infection continues to occur throughout the year or whether it returns to the usual seasonal pattern within a few years.

## Conclusions

While the seasonal trend of hMPV infection following the COVID-19 pandemic appears to have been temporarily suspended, hMPV infection can occur in any season in the post-COVID-19 era. Even in young immunocompetent adults, hMPV infection can cause pneumonia and may progress to acute respiratory failure with other bacterial co-infections.

Conversely, it is clinically important to pay attention to the involvement of hMPV and other respiratory viruses in pneumonia cases that initially appear to be bacterial based on CT findings. We believe that the MN-PCR test using a good-quality sputum sample is valuable for identifying these co-infections in pneumonia cases, and further studies are warranted to affirm that it should be covered by Japanese medical insurance.
